# Non-participation in breast cancer screening among previous cancer patients

**DOI:** 10.1007/s00432-018-2734-1

**Published:** 2018-08-10

**Authors:** Line Flytkjær Virgilsen, Anette Fischer Pedersen, Berit Andersen, Peter Vedsted

**Affiliations:** 1 Research Unit for General Practice and Section for General Medical Practice, Department of Public Health, Aarhus University, Bartholins Allé 2, Aarhus C, 8000 Aarhus, Denmark; 2 Research Centre for Cancer Diagnosis in Primary Care (CaP), Department of Public Health, Aarhus University, Bartholins Allé 2, Aarhus C, 8000 Aarhus, Denmark; 3 Department for Public Health Programmes, Randers Regional Hospital, Skovlyvej 1, 8930 Randers, Denmark; 4 Department of Clinical Medicine, Aarhus University, Palle Juul-Jensens Boulevard 82, 8000 Aarhus, Denmark

**Keywords:** Denmark, Breast cancer, Screening participation, Screening behaviour, Previous cancer patients, Cancer survivors

## Abstract

**Purpose:**

Breast cancer can be detected at early stages through organised screening. This study explored reasons for non-participation in breast cancer screening among previous cancer patients, who have high risk of developing a new primary cancer.

**Method:**

We conducted a population-based historical cohort study, including all women invited to the first organised screening round in 2008–2009 in the Central Denmark Region (*n* = 149,234). All data were based on national registers.

**Results:**

Among women with previous cancer (*n* = 6638), 25.3% did not participate in breast cancer screening compared to 20.9% of women with no registrations of previous cancer, thus previous cancer patients were 21% less likely not to participate in breast cancer screening (PRR 1.21, 95% CI 1.16–1.27). Further analysis showed that this association was due to women receiving current cancer treatment or being in palliative care in the time leading up to screening. Women with previous malignant melanoma or colorectal cancer were more likely to participate in breast cancer screening, whereas women with previous gynaecological or “other” cancer types were less likely to participate.

**Conclusion:**

Screening for breast cancer may help diagnose breast cancer at an early stage. Women with previous cancer who are not undergoing current treatment or in palliative care have the same propensity to participate as other women invited to breast cancer screening. Women with previous gynaecological cancer were less likely to participate in breast cancer screening than women with other cancer types. These results may only be generalised to similar health care systems.

## Introduction

Increasing cancer incidence and improved cancer treatment have resulted in a higher proportion of cancer survivors (Bray and Moller 2006; Engholm et al. 2010). Compared to the overall population, cancer survivors (i.e. previous cancer patients) have a higher risk of new primary cancer (Ng and Travis 2008; Soerjomataram and Coebergh 2009). They also face the risk of recurrence as well as physical- and psychological late effects from the primary cancer (Harrington et al. 2010; Hassett et al. 2014; Hovaldt et al. 2015). Underuse of secondary prevention has been observed in this group (Earle and Neville 2004), but mixed findings have been reported for breast cancer screening behaviour in cancer survivors. Some studies have found that female cancer survivors are more likely to attend breast screening (Duffy et al. 2006; Schumacher et al. 2012; Trask et al. 2005), others report that they are less likely (Jensen et al. 2015b; Schapira et al. 2000), and some show no association (Heflin et al. 2002; Manjer et al. 2015; Mayer et al. 2007).

Breast cancer screening can detect cancer at an earlier stage, which has been associated with better prognosis (Independent and Panel on Breast Cancer Screening 2012; Vejborg et al. 2011). In Denmark, breast cancer screening is freely and universally available to women aged 50–69 years (Vejborg et al. 2011). Participation in breast cancer screening is generally high in Denmark (~ 80%) (Mikkelsen et al. 2016). However, Danish studies have found that non-participation is not equally distributed as a higher proportion of non-participation has been found in specific population groups, e.g. women with lower socio-economic position (SEP) or chronic disease (Jensen et al. 2012, 2015a, b; von Euler-Chelpin et al. 2008). In a previous study, we found that women with a history of cancer (excluding breast cancer) had an up to 50% increased likelihood of non-participation in breast cancer screening (Jensen et al. 2015b). The reason for this is unclear, but it could be related to poor health status among cancer survivors or to possible misconceptions in this group; for example that enrolment in a cancer follow-up programme facilitates early detection of other cancer disease.

The aim of this study was to explore the increased likelihood of non-participation among previous cancer patients other than breast cancer. It was hypothesised that “time since diagnosis”, “ongoing cancer treatment”, “attending cancer follow-up programme other than for breast cancer” or “being in palliative care” could explain higher likelihood of non-participation among cancer survivors. The study further aimed to explore if women with specific cancer types were more likely not to participate in screening.

## Methods

### Setting and population

The study was conducted in the Central Denmark Region, where the first organised breast cancer screening round was performed in 2008–2009. The population in this region is estimated at 1.2 million. All women aged 50–69 years living in this region (*N* = 149,234) were invited by postal mail to participate in the screening programme. The invited women received a fixed (yet changeable) date, and no reminders were sent to non-participants. In total, 78.9% participated in the first screening round (Jensen et al. 2012). The age-standardised incidence rate of breast cancer was 155 per 100,000 women in 2008 (National Board of Health 2009).

### Study design and exclusion criteria

This observational register-based historical cohort study was based on data from women invited to the first organised breast cancer screening programme in the Central Denmark Region. We studied factors associated with non-participation in breast cancer screening among women with a previous cancer diagnosis (excluding breast cancer). Previous cancer was defined as a diagnosis of cancer recorded in the Danish Cancer Register (DCR) prior to the scheduled screening date (excluding non-melanoma skin cancer). The DCR holds information on all cancer cases in Denmark since 1943 (Gjerstorff 2011). We excluded 4646 women with breast cancer (ICD-10: C50) because many in this group were enrolled in a cancer follow-up programme and were specifically asked not to participate in the organised screening programme. A total of 6638 women were registered with a previous cancer between the age of 14 and their scheduled screening date. We excluded women who had died or moved between the invitation date and the booking date (*n* = 233) and women registered with a GP outside the caption area (*n* = 91). A total of 144,269 women were finally included in the analyses.

### Registers and variables

All contacts to the Danish health care system are registered in national registers, and all Danes are assigned a unique and permanent 10-digit personal Civil Registration Number (CRN) (Pedersen 2011). The CRN was used to link data on screening participation, cancer diagnosis, treatments, comorbidity and SEP.

The outcome was defined as participation in the first organised breast cancer screening round, and eligible women were categorised into “non-participation” and”participation” on the basis of registrations in an administrative database.

The following variables were defined based on data from the Danish National Patient Register (NPR) with information on all hospital contacts (Lynge et al. 2011): Previous cancer patients were categorised as undergoing “*current cancer treatment*” if registered with chemotherapy or radiotherapy in the NPR within 6 months prior the scheduled screening date. They were also considered to undergo treatment if registered with any operation related to cancer within 3 months prior the scheduled screening date (i.e. the operation was performed based on a “DC” ICD-10 diagnosis code), except for endoscopies or biopsies as the majority of these procedures are used for diagnostic purposes. *“Attending a cancer follow-up programme”* was defined based on registration in the NPR with a procedure code for cancer follow-up (SKS: DZ08 and all sub-codes) up to 2 years prior to the scheduled screening date among previous cancer patients. Previous cancer patients were considered to be in “*palliative care*” if registered with previous cancer and listed as dead in the Civil Registration System within 1 year after the scheduled date of screening. A total of 54 women were registered with “undergoing treatment”, “attending cancer follow-up” and “palliative care”. “*Time from diagnosis to the scheduled screening date*” was categorised into: 0–1 year, > 1–5 years, > 5–10 years, > 10 years.

Cancer types were stratified into gynaecological cancer (ICD-10: C51-C58), colorectal cancer (ICD-10: C18-C20), lung cancer (ICD-10: C34), haematological cancer (ICD-10: C81-C96), malignant melanoma (ICD-10: C43) and other cancer types (all other ICD-10: “DC” codes).

SEP was included using the following variables: *age* (divided into 50–54 years, 55–59 years, 60–64 years and 65–69 years), *ethnicity* (divided into Danish decedents and immigrants), *marital status* (divided into married/cohabitating or living alone) and *education* [divided into ≤ 10 years, 11–15 years or > 15 years of education in accordance with UNESCO’s classification (UNESCO 2014)]. Charlson Comorbidity Index (CCI) scores (Quan et al. 2011) were obtained from hospital contacts from the NPR of the included diseases (except for cancer) 10 years prior to the scheduled screening date and categorised into the scores 0, 1 and ≥ 2.

### Statistical analysis

Generalised linear models (GLM) with log link and Bernoulli regression models (Barros and Hirakata 2003; Zou 2004) were applied to study possible factors of importance for non-participation in breast cancer screening among previous cancer patients. Prevalence rate ratios (PRR) with 95% confidence intervals (CI) were chosen since the proportion of non-participation (the outcome) was more than 20% (Barros and Hirakata 2003; Zou 2004). To study if the increased likelihood of non-participation was explained by SEP, CCI or the severity of the previous cancer, we conducted analyses including the entire population (*n* = 144,264). Unadjusted analyses were performed to study if each variable independently was associated with non-participation. Furthermore, adjustment for socio-demographic variables and comorbidity was performed (model 1). Finally, a fully adjusted model was performed (model 2). We found a high degree of multicollinearity between the variables “previous cancer” and “time since diagnosis”. Therefore, we conducted a restricted analysis among previous cancer patients (*n* = 6638) to explore if “time since diagnosis” was associated with non-participation. Finally, to study non-participation among specific cancer groups, we restricted the analyses to previous cancer patients and excluded women undergoing current treatment and women in palliative care (*n* = 5777). Robust variance estimates were used to adjust for clustering of patients by general practice in all models (Davis 2001). All statistical analyses were conducted using Stata statistical software, version 14.

### Ethical approvals

No ethical approval was required according to Danish law and the National Committee on Health Research Ethics in the Central Denmark Region as the study was based on registry data (journal no. 181/2011). The project was approved by the Danish Data Protection Agency (journal no.: 2009-41-3471 and journal no.: 1-16-02-109-09).

## Results

### General observations on non-participation

A total of 25.3% of previous cancer patients did not participate in breast cancer screening compared to 20.9% of women with no registrations of previous cancer (Table 1).

**Table 1:** Distribution of participants and non-participants in the breast cancer screening programme according to previous cancer, socio-economic position and CCI score (*n* = 144,264, numbers vary due to missing observations)

	Participants	Non-participants	*P* value (chi^2^)
	*N* (% column)	*N* (% column)	
In total	113,811 (78.9)	30,453 (21.1)	
Previous cancer			< 0.001
No	108,856 (79.1)	28,770 (20.9)	
Yes	4955 (74.7)	1683 (25.3)	
In cancer treatment			< 0.001
No	113,486 (79.0)	30,111 (21.0)	
Yes	325 (48.3)	342 (48.6)	
In cancer follow-up programme			< 0.001
No	113,368 (78.9)	30,273 (21.1)	
Yes	443 (71.1)	180 (28.9)	
In palliative care			< 0.001
No	113,703 (79.0)	30,213 (21.0)	
Yes	108 (31.0)	240 (69.0)	
Time from diagnosis to screening date (in years)			< 0.001
No diagnosis	108,856 (79.1)	28,770 (20.9)	
0–1	403 (58.7)	284 (41.3)	
> 1–5	1152 (73.4)	417 (26.6)	
> 5–10	915 (77.9)	259 (22.1)	
> 10	2485 (77.5)	723 (22.5)	
Age on the screening date (in years)			< 0.001
50–54	30,965 (80.4)	7536 (19.6)	
55–59	30,722 (80.2)	7580 (19.8)	
60–64	30,532 (79.2)	7998 (20.8)	
65–69	21,592 (74.6)	7339 (25.4)	
Marital status			< 0.001
Married/cohabiting	88,590 (82.7)	18,484 (17.3)	
Living alone	25,183 (67.9)	11,924 (32.1)	
Ethnicity			< 0.001
Danish descendant	110,018 (79.6)	28,201 (20.4)	
Immigrant	3773 (62.9)	2224 (37.1)	
Education (years)			< 0.001
≤ 10	39,214 (75.6)	12,651 (24.4)	
11–15	47,661 (81.8)	10,624 (18.2)	
> 15	25,549 (80.2)	6286 (19.8)	
CCI score			< 0.001
0	94,532 (80.0)	23,584 (19.9)	
1	11,730 (76.7)	3568 (23.3)	
≥ 2	7549 (69.6)	3301 (30.4)	

*CCI* Charlson’s comorbidity index

The proportion of non-participation was higher among women diagnosed with cancer within 1 year of the scheduled screening date, in current cancer treatment, in cancer follow-up and in palliative care. The proportion of non-participation was also higher in women of older age, living alone, of immigrant descent, with low education and a CCI score of ≥ 2 (Table 1).

### Non-participation among previous cancer patients

Women with previous cancer were 21% less likely to participate in screening compared with women with no registrations of previous cancer (PRR = 1.21, 95% CI 1.16–1.27). This remained statistically significant when adjusted for SEP and CCI [PRR = 1.14 (95% CI 1.09–1.19)] (model 1, Table 2). After further adjustments for current cancer treatment, cancer follow-up and palliative care, no association was seen between previous cancer and participation in breast cancer screening (model 2, Table 2).

**Table 2:** The association between non-participation in breast cancer screening and previous cancer patient in cancer treatment, in cancer follow-up or in palliative care at the time of the scheduled screening date (*n* = 144,264)

	*n*	Unadjusted PRR (95% CI)	Model 1^a^ PRR (95% CI)	Model 2^b^ PRR (95% CI)
Previous cancer				
No	137,626	1 (ref.)	1 (ref.)	1 (ref.)
Yes	6638	**1.21 (1.16–1.27)**	**1.14 (1.09–1.19)**	0.99 (0.95–1.04)
In cancer treatment				
No	143,597	1 (ref.)	1 (ref)	1 (ref.)
Yes	667	**2.45 (2.27–2.34)**	**2.29 (2.12–2.48)**	**1.75 (1.57–1.96)**
In cancer follow-up				
No	143,641	1 (ref.)	1 (ref.)	1 (ref.)
Yes	623	**1.37 (1.22–1.54)**	**1.29 (1.16–1.43)**	0.95 (0.84–1.08)
In palliative care^c^				
No	143,916	1 (ref.)	1 (ref.)	1 (ref.)
Yes	348	**3.28 (3.05–3.53)**	**2.80 (2.59–3.01)**	**1.95 (1.75–2.18)**

Significant values are in bold

^a^Model 1: adjusted for age, ethnicity, marital status and education and CCI score

^b^Model 2: adjusted for variables in model 1 and “cancer treatment”, “cancer follow-up” and “palliative care”

^c^ Died within 12 months after scheduled screening date

Being in palliative care was associated with non-participation (PRR_adj_ = 1.95, 95% CI: 1.75–2.18). The same was found for undergoing cancer treatment (PRR_adj_ =1.75, 95% CI 1.57–1.96) (Table 2). Women diagnosed with cancer up to 5 years prior to the scheduled screening date were statistically significantly more likely not to participate compared with women diagnosed more than 10 years prior to the scheduled screening date (Table 3, model 1). However, this propensity disappeared when adjusting for current cancer treatment, cancer follow-up and palliative care (Table 3).

**Table 3:** The association between non-participation in breast cancer screening and time from diagnosis to scheduled screening date among previous cancer patients (n = 6638)

	*n*	Unadjusted PRR (95% CI)	Model 1^a^ PRR (95% CI)	Model 2^b^ PRR (95% CI)
Time from diagnosis to screening (in years)				
> 10	3208	1 (ref.)	1 (ref.)	1 (ref.)
> 5–10	1174	0.98 (1.87–1.10)	1.00 (0.89–1.12)	0.97 (0.87–1.09)
> 1–5	1569	**1.18 (1.07–1.30)**	**1.18 (1.06–1.30)**	1.00 (0.90–1.12)
0–1	687	**1.83 (1.63–2.06)**	**1.80 (1.60–2.01)**	1.07 (0.93–1.23)

Significant values are in bold

^a^Model 1: Adjusted for age, ethnicity, marital status, education and CCI score

^b^Model 2: Adjusted for variables in model 1 and “cancer treatment”, “cancer follow-up” and “palliative care”

### Non-participation among subgroups of cancer patients

A total of 5850 previous cancer patients were not undergoing treatment or palliative care in the time leading up to the scheduled screening date (Table 4). In the adjusted analyses, women with previous gynaecological cancer or “other cancer types” were more likely not to participate, whereas women with colorectal cancer or malignant melanoma were more likely to participate (Table 4).

**Table 4:** The association between specific cancer groups and non-participation in breast cancer screening among previous cancer patients excluding those undergoing cancer treatment or was in palliative care at the time of scheduled screening date (*n* = 5580)

	*n*	Unadjusted PRR (95%CI)	Model 1 PRR (95%CI)
Gynaecological cancer			
No	4190	1 (ref.)	1 (ref.)
Yes	1605	1.10 (1.00–1.23)	**1.12 (1.01–1.24)**
Colorectal cancer			
No	5120	1 (ref.)	1 (ref.)
Yes	665	**0.79 (0.66–0.94)**	**0.82 (0.69–0.96)**
Haematological cancer			
No	5538	1 (ref.)	1 (ref.)
Yes	240	1.11 (0.89–1.37)	1.09 (0.87–1.35)
Lung cancer			
No	5565	1 (ref.)	1 (ref.)
Yes	218	**1.38 (1.11–1.72)**	1.18 (0.94–1.48)
Malignant melanoma			
No	4980	1 (ref.)	1 (ref.)
Yes	808	**0.58 (0.48–0.71)**	**0.66 (0.54–0.80)**
Other cancer types			
No	4773	1 (ref.)	1 (ref.)
Yes	1019	**1.17 (1.03–1.33)**	**1.16 (1.02–1.32)**

Significant values are in bold

Model 1: Each cancer type is adjusted for age, ethnicity, marital status, education and CCI score

## Discussion

### Main findings

Women with previous cancer were generally less likely to participate in breast cancer screening, but the association between previous cancer and non-participation disappeared when accounting for women undergoing current cancer treatment and women in palliative care in the time leading up to the screening. Women with colorectal cancer or malignant melanoma were more likely to participate in the screening, whereas women with gynaecological cancer or “other cancer types” were less likely to participate.

## Strengths and limitations

The study population comprised all women invited to the first organised screening round in the Central Denmark Region in 2008–2009. Complete data for the entire prevalent screening population were obtained from an administrative database, and precise exclusion of relevant groups was possible. Selection bias is, therefore, minimal.

Linkage of valid register-based and almost complete data on death, cancer disease, comorbidity, socio-demographic variables and hospital contacts (Gjerstorff 2011; Lynge et al. 2011) was possible on an individual level using the unique CRN (Pedersen 2011). Thus, information bias is also of minimal concern.

To identify cancer treatment, we developed an algorithm identifying cancer-specific surgery in the NPR up to 3 months prior to the scheduled screening date. The NPR does not explicitly state if a given procedure is intended as a diagnostic precaution or performed as part of treatment. We excluded all biopsies and endoscopies as they are usually used for diagnostic purposes. However, endoscopies are sometimes performed as treatment for some malignancies. Thus, we might falsely have excluded some cancer operations. This would result in bias towards the null, as women undergoing cancer treatment were more likely not to participate.

### Generalisability of findings

The results of this study may be generalised to other regions of Denmark and other countries with similar organisation of the health care system. We do not know whether our results for breast cancer screening can be applied to other types of screening programmes, e.g. colorectal cancer screening, but it seems plausible that ongoing treatment and palliative care would show similar effects in other cancer populations.

### Interpretation and comparison with other research

Besides a high risk of multiple chronic diseases (Hovaldt et al. 2015), patients with previous cancer have a high risk of developing new cancers (Ng and Travis 2008; Soerjomataram and Coebergh 2009). Therefore, high coverage in the screening programme could be even more important in this group.

This study confirms our earlier finding that previous cancer patients are more likely not to participate in breast cancer screening (Jensen et al. 2015b). However, a detailed analysis of factors that might explain this association revealed that the association was primarily driven by non-participation among women receiving current cancer treatment or was in palliative care in the time leading up to the screening.

Previous research has reported inconclusive findings as to whether previous cancer patients are less likely to participate in breast cancer screening (Duffy et al. 2006; Jensen et al. 2015b; Mayer et al. 2007; Schapira et al. 2000; Schumacher et al. 2012; Trask et al. 2005), and this study might play a crucial role in explaining the contradictory findings. Our study highlights that screening participation among previous cancer patients is affected by disease severity at the time of the scheduled screening date, thus patient’s disease severity should be considered in future studies exploring this subject.

This study found that “time since diagnosis” was associated with higher non-participation only because recently diagnosed cancer patients were more often receiving treatment or in palliative care at the time of the screening. To our knowledge, only two previous studies have investigated time since diagnosis and non-participation among previous cancer patients. Both of these studies found that participation decreased with the time since diagnosis (Doubeni et al. 2006; Snyder et al. 2009). As these studies only included breast cancer patients, the results are difficult to compare. One explanation for the differing results could be that we included the full period from a previous cancer diagnosis, whereas the other studies did not assess screening participation before end of treatment (Doubeni et al. 2006) or 1 year after diagnosis (Snyder et al. 2009).

Being enrolled in a cancer follow-up programme may lead to the misconception that being followed up for another cancer would also provide early detection of breast cancer. Such potential misconception was not confirmed in this study, which could indicate that these women attend breast cancer screening as intended. Previous studies have found that continuous contacts with an oncologist or primary physicians increases the likelihood of attending regular breast cancer screening in patients with previous breast cancer (Earle et al. 2003; Wirtz et al. 2014) and other previous cancer types (Bellizzi et al. 2005).

This study found differences in the likelihood of non-participation across selected groups of cancer patients. Previous colorectal cancer patients were more likely to participate in the screening programme. This is somewhat in line with our previous findings that women with chronic bowel diseases are more likely to participate in screening (Jensen et al. 2015b). In addition, women with previous malignant melanoma had increased likelihood of participating in breast cancer screening. This group has been shown to have higher socio-economic position (Birch-Johansen et al. 2008), and this could be a possible explanation. However, the association was still significant after adjustment for socio-demographic variables and comorbidity. Differences in health behaviour and awareness of the importance of early cancer diagnosis could also explain the increased participation in this group. These women may also have first-hand experience with potentially serious illness without clear physical symptoms, and this could increase screening participation. Contrary to expectations, previous gynaecological cancer patients were less likely to participate in screening, which calls for further research to explain the possible mechanisms.

## Conclusion

In this study, we found that non-participation in breast cancer screening was mainly driven by non-participation among women receiving current treatment or palliative care. Taking this into account, women with a previous cancer attended breast cancer screening similarly to other women. Previous malignant melanoma and colorectal cancer patients were more likely to participate, whereas previous gynaecological cancer patients and women with “other cancers” were less likely to participate.

## Abbreviations

CCI: Charlson’s Comorbidity Index
CI: Confidence interval
CRN: Civil registration number
DCR: Danish Cancer Register
GLM: Generalised linear models
ICD-10: International Classification of Diseases, 10th version
NPR: National Patient Register
PRR: Prevalence rate ratio
